# Holistic Approaches in Endometriosis - as an Effective Method of Supporting Traditional Treatment: A Systematic Search and Narrative Review

**DOI:** 10.1007/s43032-024-01660-2

**Published:** 2024-07-23

**Authors:** Agnieszka Mazur-Bialy, Sabina Tim, Anna Pępek, Kamila Skotniczna, Gabriela Naprawa

**Affiliations:** 1 Department of Biomechanics and Kinesiology, Faculty of Health Science, Jagiellonian University Medical College, Skawińska 8, Krakow, 31-066 Poland; 2 Student Scientific Group, Faculty of Health Science, Jagiellonian University Medical College, Krakow, Poland

**Keywords:** Endometriosis, Physiotherapy, Physical activity, Manual therapy, Quality of life, Pain

## Abstract

Endometriosis is one of the gynecological diseases where women suffer from pain, quality of life decreased. The aim of this review was to describe the most common non-medical methods used in the treatment of symptoms associated with endometriosis and to determine their effectiveness. The review was performed in PubMed, Embase and Web of Science databases. Randomized controlled trials, case studies, observational studies, retrospective studies, prospective studies, pilot studies, trails, publications in English or Polish were searched based on the Participant-Intervention-Comparator-Outcomes-Study design (PICOS) format. The criteria used to select studies were: women with endometriosis, no cancer, included any physiotherapeutic or non-medical intervention. 3706 articles were found, however only 26 met the inclusion criteria and were included in the review. Quality of the studies was assessed by Risk of Bias 2 tool and ROBINS-1 tool. The most holistic approach used in the treatment of symptoms of endometriosis include physical therapy, manual therapy, electrophysical agents acupuncture, diet and psychological interventions. Most research has focused on relieving pain and increasing quality of life. Non-medical methods showed reduction of symptoms of endometriosis. Physical activity, manual therapy, electrophysical agents, acupuncture, diet and cognitive behavioral therapy showed no negative side effects and reduced pain, what improved the quality of life and reduced the perceived stress.

**Supplementary Information:**

The online version contains supplementary material available at 10.1007/s43032-024-01660-2.

## Introduction

Endometriosis is a gynecological condition characterized by the presence of estrogen-sensitive tissue resembling the endometrium found outside the uterus [1]. Endometrial glands are typically observed in the pelvic region, including the ovaries, ligaments, peritoneum, intestines, bladder, lymph nodes, and even the lungs, diaphragm, or pericardium [2, 3]. It is estimated that approximately 15% of women of reproductive age experience endometriosis [4]. However, diagnostic difficulties and variations in prevalence across different populations may disturb the correct result of the frequency of this disease [5].

Symptoms of endometriosis vary widely. Some women may remain asymptomatic for years, while others may experience painful menstruation, intermenstrual bleeding, infertility, urinary issues, painful intercourse, painful bowel movements, diarrhoea, or non-menstrual abdominal pain [2, 6, 7]. Nevertheless, the predominant symptom is pain, which can be nociceptive, inflammatory or neuropathic [8]. Chronic pain can lead to central sensitization, which makes pain management difficult, which is noticeable in the population of women with endometriosis [9].

The pain associated with endometriosis involves a complex interplay between peripheral nerve conduction, the peritoneum, and the central nervous system. Increased presence of small unmyelinated nerve fibers and neurotrophic factors near endometriotic lesions suggests their role in pain development. Furthermore, ongoing inflammatory processes cause the release of pro-inflammatory molecules by sensory fibers, which also contributes to increased pain perception. Nerve fiber sensitization due to the pro-inflammatory environment increases pain sensitivity. An impaired immune response to endometrial cells and tissues in patients with endometriosis may contribute to the growth and attachment of endometrial cells, which further worsens pain [8, 10, 11].

Aside from diminishing quality of life, pain contributes to myofascial changes, leading to improper body posture, weakened trunk muscle function, altered spine curvature, and decreased lung function [12, 13]. Patients with endometriosis exhibit thinner abdominal wall muscles, decreased lumbopelvic stability and less resistance in trunk flexor and extensor muscles [14]. Incorrect body posture affects the pelvic floor as well, manifesting as pelvic floor hyperactivity among women with endometriosis, resulting in sexual dysfunction and other pelvic floor dysfunctions like urinary incontinence or constipation [15].

The primary objective of non-medical methods in managing endometriosis is pain relief and pelvic floor function improvement [16], along with post-surgical support [17]. This techniques aim to relax muscles, reduce inflammation, and disrupt the pain cycle, ultimately enhancing quality of life [18]. The aim of this review was to describe the most common physiotherapeutic and non-medical methods used in the treatment of symptoms associated with endometriosis and to determine their effectiveness.

## Materials and Methods

The study protocol was prepared following the guidelines of the Preferred Reporting Items for Systematic Review and Meta-Analysis (PRISMA) [19]. The research protocol has been approved by PROSPERO no. CRD42023389400 “Physiotherapy in endometriosis - as an effective method of supporting traditional treatment: a systematic review.“ Inclusion criteria were formulated based on the Participant-Intervention-Comparator-Outcomes-Study design (PICOS) format.

Participants: women with endometriosis, no cancer.

Intervention: any physiotherapeutic or non-medical intervention (exercise, manual therapy, physical therapy, diet, psychologic intervention).

Comparasion: no intervention, placebo.

Outcomes: therapy effectiveness assessment, quality of life, pain, pelvic floor, sex life, muscle function.

Study design: studies in Polish and English, no time limit, pilot study, randomized control trial, prospective study, retrospective study, observational study.

The search process involved four researchers independently scouring databases including Medline-Pub Med, Embase, and Web of Science. The following phrase was used to search for articles: endometriosis and (physiotherapy or rehabilitation or electrotherapy or electrophysical agents or exercise or yoga or visceral therapy or acupuncture or manual therapy or physical therapy or massage or trigger points or breathing or biopsychosocial or mindfulness or relaxation or complementary therapy or holistic approach).

Titles and abstracts were initially screened for relevance, with subsequent inclusion of studies addressing endometriosis symptoms such as pain, quality of life, physical function, and infertility. Exclusion criteria comprised studies not published in English or Polish, those describing surgical procedures or animal models, and those involving pediatric or male populations, or primarily mathematical analyses. Discrepancies in study selection were resolved through consensus.

After initial screening, full-text versions of selected articles were obtained and scrutinized for study type, participant demographics, intervention details, outcome assessment methods, questionnaires utilized, and main findings, ensuring alignment with inclusion criteria.

Risk of bias assessment was conducted independently by two researchers using the Risk of Bias 2 tool [20] for randomized studies and ROBINS-I [21] for non-randomized trials, evaluating various domains such as randomization process, handling of missing data, intervention adherence, outcome measurement, and reporting integrity to determine overall study risk levels.

## Results

### Characteristics of the Studies

**Fig. 1:**
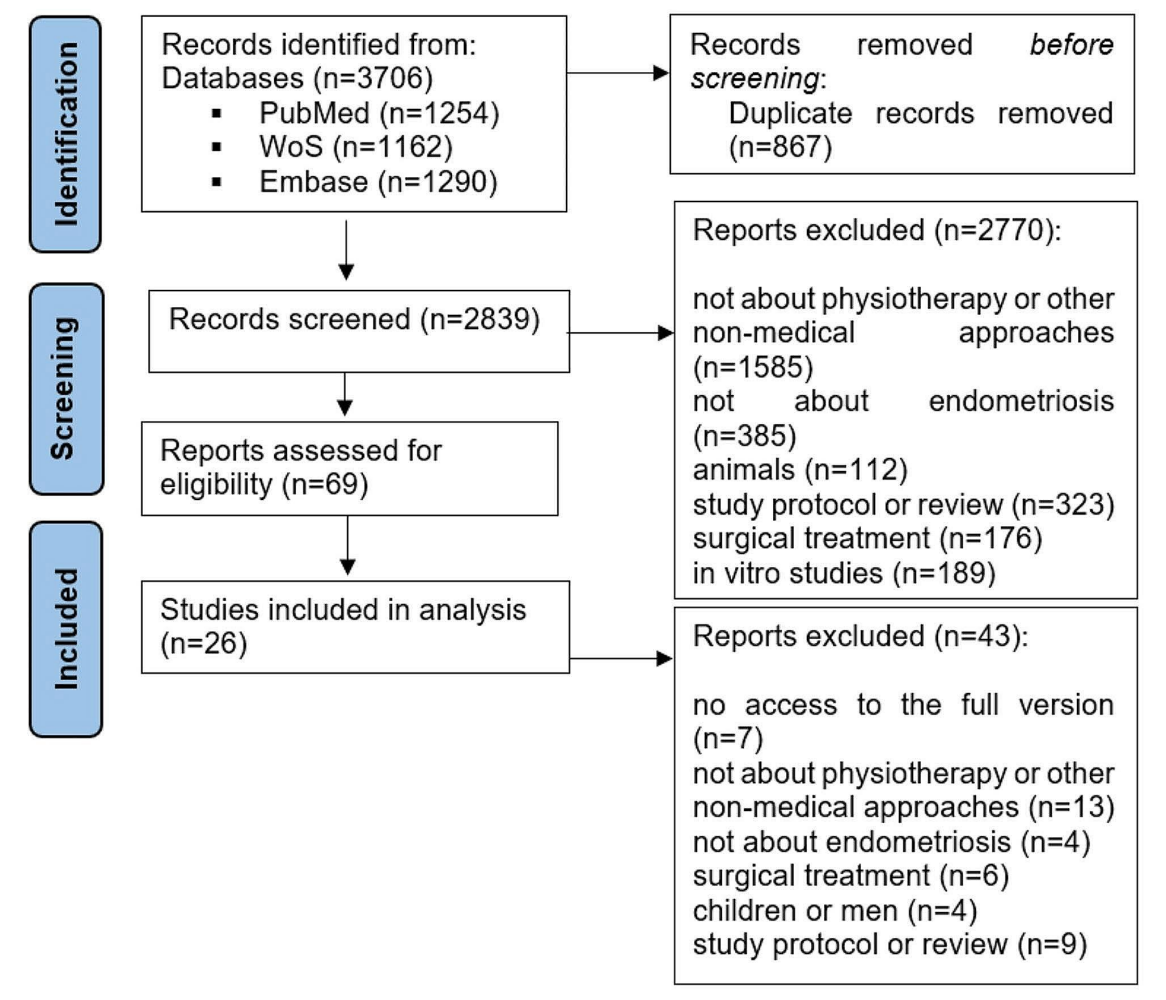
PRISMA diagram

Based on the phrases presented, a total of 3706 works were found. After removing duplicates, 2839 works remained. After analyzing the titles and abstracts, 2770 works were rejected. There were 69 works left to be fully read. 26 works met the final inclusion criteria. A detailed analysis of the individual stages of the review is presented in the PRISMA diagram (Fig. 1).

The studies included into the analysis assess pain (19 studies), quality of life (17 studies), mental health (9 studies), stress (6 studies), dyspareunia (7 studies) among women with endometriosis. The physiotherapeutic interventions that have been described are: physical activity, manual therapy, acupuncture, and physical therapy. The characteristics of the studies included in the review are presented in Table 1.

**Table 1:** Characteristics of the included studies divided into areas under observation and treatment of patients with endometriosis

Author, year	Areas under observation					Therapy						
	Pain	QoL	Mental Health	Stress	Dyspareunia	PA	MT	EPA	AP	SE	Diet	CBT
Goncalves, 2016 [22]	+	+				+						
Goncalves, 2016 [23]	+		+	+		+						
Petrelluzzi, 2012 [24]	+		+	+		+		+				
Armour, 2019 [25]		+	+	+		+		+				
Bergstrom, 2005 [26]						+				+		
Merlot, 2022 [27]	+							+				
Zhao, 2011 [28]		+	+			+				+		
Darai, 2014 [29]	+	+					+					
del Forno, 2020 [30]	+				+		+	+				
Wurn, 2011 [31]	+				+		+					
Bi, 2018 [32]	+	+	+					+				
Hawkins, 2003 [33]	+	+						+				
Mira, 2020 [34]	+	+			+			+				
Thabet, 2018 [35]	+	+						+				
Rubi Klein, 2010 [36]	+								+			
Muñoz-Gómez, 2023 [37]	+	+	+	+			+					
de Sousa, 2016 [38]	+	+			+				+			
Sillem, 2016 [39]	+						+					
Tian, 2022 [40]	+	+							+			
del Forno, 2024 [41]					+		+	+				
Nodler, 2020 [42]	+	+	+								+	
Cirillo, 2023 [43]	+	+			+						+	
van Haaps, 2023 [44]	+	+			+						+	
Donatti, 2024 [45]		+		+								+
Wu, 2022 [46]		+	+	+								+
Kold, 2012 [47]		+	+									+

QoL– quality of life; PA– physical activity; MT– manual therapy; EPA– electrophysical agents; AP- acupuncture; SE– alleviating the side effects of medications; CBT– Cognitive-Behavioural Therapy

**Fig. 2:**
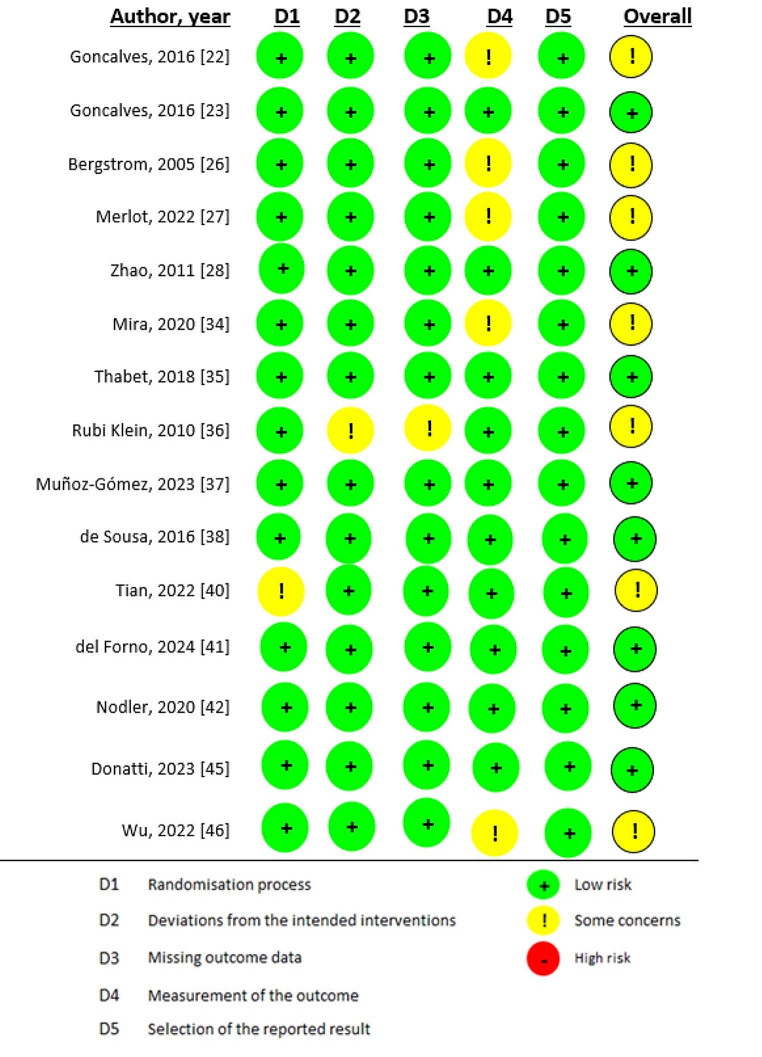
Risk of bias of randomized clinical trials assessed in RoB-2 tool

Risk of Bias of fifteen studies were assessed using RoB 2 tool. Eight studies were assessed as low risk of bias, then seven studies were assessed by moderate risk of bias. Eleven studies were assessed using ROBINS-I tool. Four studies have low risk of bias, seven studies have moderate risk of bias. See Fig. 2; Table 2.

**Table 2:** ROBINS analysis of included studies of physiotherapy techniques used in reduce symptoms of endometriosis

Author, year	Bias due to confunding	Bias in selection of participants into the study	Bias due to Missing data	Bias in measurement of outcomes	Bias in selection of the reporter result	Overall
Petrelluzzi, 2012 [24]	Low	Low	Low	Low	Low	Low
Armour, 2019 [25]	Moderate	Moderate	Low	Low	Low	Moderate
Darai, 2014 [29]	Moderate	Moderate	Low	Low	Low	Moderate
Del Forno, 2020 [30]	Moderate	Moderate	Low	Moderate	Moderate	Moderate
Wurn, 2011 [31]	Low	Low	Low	Low	Low	Low
Bi, 2018 [32]	Low	Low	Low	Low	Low	Low
Hawkins, 2003 [33]	Moderate	Moderate	Moderate	Low	Low	Moderate
Sillem, 2016 [39]	Moderate	Moderate	Moderate	Moderate	Moderate	Moderate
Cirillo, 2023 [43]	Low	Low	Low	Low	Low	Low
van Haaps, 2023 [44]	Moderate	Low	Low	Low	Low	Moderate
Kold, 2012 [47]	Moderate	Low	Low	Low	Low	Moderate

### Physical Activity in the Treatment of Endometrial Symptoms

According to Piggin [48], ‘physical activity involves people moving, acting and performing within culturally specific spaces and contexts, and influenced by a unique array of interests, emotions, ideas, instructions and relationships.’ Research on physical activity (PA) among women with endometriosis focuses on alleviating the side effects of drugs and reducing pain while improving quality of life. PA utilized in treating endometriosis symptoms includes breathing exercises, yoga, Pilates, muscle relaxation, and aerobic activities.

Armour et al. [25] estimated that exercises, yoga, Pilates, stretching, and breathing were among the self-management strategies adopted by women with endometriosis. However, women rated these interventions as less effective in reducing pain compared to cannabis, heat, diet, or acupressure. Nonetheless, physical interventions reduced pain on average from 4.5 to 4.9 points on a 0–10 scale (0 being ineffective; 10 being extremely effective). Conversely, after engaging in physical activity, women reported increased pelvic pain (especially cramp pain) and fatigue, particularly after Pilates practice [25].

One of the mind-practice activities is yoga, which integrates meditation, physical exercises, and breathing techniques. Two-hour yoga sessions twice a week in women with endometriosis reduced pain and improved quality of life; however, yoga did not affect or decrease menstrual blood flow [22]. It was emphasized that yoga techniques, particularly breathing exercises, were beneficial in coping with pain, leading to reduced reliance on painkillers. Yoga also fostered self-control, self-awareness, autonomy, improved sleep, better management of panic attacks, and increased self-confidence [23]. Ten sessions combining breathing exercises, individualized stretching and strengthening exercises, massage, transcutaneous electrical nerve stimulation, and psychological intervention reduced salivary cortisol levels, thereby decreasing perceived stress levels and enhancing quality of life [24].

Many women use pharmacotherapy to alleviate pain, which may have side effects. PA may play a role in mitigating these effects. Three 30-minute brisk walking sessions and two 1-hour aerobic training sessions per week reduce bone density loss among women with endometriosis undergoing pharmacological treatment with gonadotropin-releasing hormone [26]. Pharmacological treatment can be complemented by progressive muscle relaxation to reduce pain and the side effects of hormone treatment. After 12 weeks, attending group classes twice a week with Jacobson’s relaxation concept and home practice significantly improved overall quality of life [28].

PA is often chosen as a self-management strategy for addressing endometriosis symptoms. It enhances quality of life, reduces pain, and mitigates the effects of pharmacological treatment. The characteristics of studies elucidating the effectiveness of physical activity in treating endometriosis symptoms are presented in Table 3.

**Table 3:** Characteristics of studies on the effectiveness of the use of physical activity in the treatment of endometrial symptoms

Author, year, country	Purpose	Participants	Intervention	Results
Goncalves et al. 2016 Brazil [22]	Assessment of the effects of yoga on quality of life, the severity of chronic pelvic pain and the menstrual cycle.	*n* = 40 women Yoga group: *n* = 28 Mean age: 34.5 ± 7.4 Non-yoga group: *n* = 12 Mean age: 35.75 ± 4.7	Yoga group: 2-h yoga session twice a week for 8 weeks Non-yoga group: no intervention Assessment: VAS, EHP-30	Reduction of chronic pelvic pain (*p* = 0.0046) and improvement in QoL after yoga practice.
Goncalves et al. 2016, Brazil [23]	Assessment of the mental and emotional attitude to yoga, pain management after the intervention, and peripheral benefits.	*n* = 15 women aged 24–49 years	Interview after completing yoga sessions, questions about expectations regarding the practice of yoga, pain management, physical and emotional stage, benefits of yoga.	Women have reported benefits of yoga in pain control through breathing control, increased self-awareness, autonomy, self-care, and reduced use of painkillers.
Petrelluzzi et a. 2012, Brazil [24]	Assessment of the influence of the mind-body relation on the perceived by women levels of stress, pain, HRQL and activity of the hypothalamic-pituitary-adrenal axis in women with endometriosis and chronic pelvic pain.	*n* = 30, completed *n* = 26 mean age = 32.2 ± 1.3	10 sessions of physical therapy, breathing therapy, stretching, TENS, and psychological intervention, one session per week for 10 weeks. Assessment: PSQ, SF-36, VAS, salivary cortisol levels	Physical and psychological therapies were effecting in reducing pain, stress (*p* < 0.05) and normalized cortisol levels (*p* < 0.05).
Armour et al. 2019, Australia [25]	Assessment of self-management coping with pain in endometriosis and their effectiveness.	*n* = 484 women with endometriosis mean age = 31 ± 7.4	Online survey, questions about self-management strategies with endometriosis.	Self-care and lifestyle choices were commonly used by women with endometriosis. Cannabis was the most effective in pain reduction.
Bergstrom et al. 2005, Sweden [26]	Assessment of the effect of exercise on bone mineral density in hormone-treated women with endometriosis.	*n* = 19 women Exercise group: *n* = 8 Mean age = 27.04 ± 4.39years Control group: *n* = 11 Mean age = 31.27 ± 5.04 years	Exercise group: 30-min fast walks and two 1-h aerobic training per weeks for 12 months Control group: No change in lifestyle Assessment: bone mineral density	Less decrease in bone density in the exercise group (0.6%) compared to control group (3.6%) (*p* = 0.029).
Zhao et al. 2012, China [28]	Assessment of the impact of PRM training on depression and quality of life in patients with endometriosis treated with hormones.	*n* = 100 women Progressive muscle relaxation (PMR) group: *n* = 50, completed *n* = 42 Control group: *n* = 50, completed *n* = 45	PMR group: Twenty-four 40-min group PMR practice sessions over 12 weeks, twice per week and one dose of depot leuprolide Control group: one dose of depot leuprolide Assessment: STAI, HADS-D, SF-36	Improving the results of anxiety, depression and overall quality of life in the PMR group (*p* < 0.05).

HADS-D– Hospital Anxiety and Depression Scale; PRM–Progressive Muscle Relaxation; STAI– State-Trait Anxiety Inventory; VAS - Visual Analogue Scale; EHP- Endometriosis Health Profile; PSQ- Perceived Stress Questionnaire; SF-36- 36-Item Short-Form Health Survey; QoL– Quality of Life; TENS - transcutaneous electrical nerve stimulation

### Manual Therapy in the Treatment of Endometrial Symptoms

Manual therapy, a treatment method employed by physical therapists, involves a hands-on approach. It encompasses techniques such as joint and soft tissue mobilization, stretching, and acupressure, which have the potential to alleviate pain [49].

Muñoz-Gómez et al. [37] performed comprehensive techniques, including spinal and sacroiliac manipulation, mobilization of the abdominal and broad ligaments, and pelvic diaphragm release. Their manual therapy intervention resulted in a 30.76% reduction in pain after six weeks and led to improvements in control, powerlessness, and emotional well-being among women with endometriosis [37]. Many women with endometriosis have increased pelvic floor muscle tone [30] and adhesions [31]. Some manual techniques can reduce tissue thickening and adhesion, leading to improved mobility in the area, as well as reducing pain, improving quality of life, dyspareunia and dysmenorrchea, as confirmed by Wurn et al. [31]. Certain manual techniques have been shown to decrease tissue thickening and adhesions, thereby enhancing mobility and reducing pain, improving quality of life, dyspareunia, and dysmenorrhea, as confirmed by Wurn et al. [31]. Their intensive therapy sessions, initially lasting 2 h per week for 5 months and progressing to sessions lasting up to 4 h per day for 5 days, resulted in improvements in menstrual cycle, dysmenorrhea, and dyspareunia [31]. Manual therapy can be supported by 3D/4D transperineal ultrasound to precisely identify areas of increased tension and abnormal muscle function [30]. Del Forno et al. [30] performed five 30-minutes session of Thiele massage, supported by 3D/4D transperineal ultrasound, involving stretching and acupressure of the pelvic floor muscles to restore normal tone and induce relaxation. This therapy led to improvements in the Levator Hiatus Area and reductions in both deep and superficial dyspareunia [30]. Additionally, improvements in superficial dyspareunia, chronic pelvic pain, and pelvic floor muscle relaxation were observed after Thiele massage, although del Forno et al. [41] did not confirmed effects on urinary, bowel and sexual function [41]. Another type of manual therapy is Osteopathic Manipulative Therapy (OMT). Darai et al. [29] showed that approximately 1-hour OMT of the mobilisation of uterus, colon, the peritoneum and around the vertebrae L1 and L2 improved physical and mental quality of life women with endometriosis [29]. As well as osteopathic mobilisation of sacroiliac joints, diaphragm, abdominal organ, temporomandibular joints and cervical spine mobilisation and PFM manual relaxation improved symptoms in women with long histories of endometriosis [39].

Physiotherapists frequently employ manual therapy to address pain, with osteopathic techniques becoming increasingly popular. Properly selected techniques have the potential to alleviate pain, enhance functioning, and improve quality of life. The characteristics of studies outlining the effectiveness of manual therapy in treating endometriosis are detailed in Table 4.

**Table 4:** The characteristics of studies describing the effectiveness of manual therapy in the treatment of endometrial symptoms

Author, year, country	Purpose	Participants	Intervention	Results
Darai et al. 2015, France [29]	To assess the OMT on qol of patients with deep infiltrating endometriosis.	*n* = 20 women (15 completed) Median age = 30.4	OMT including mobilization of the uterus, peritoneal mobility, the colon, and L1 and L2 for median time 60 min (range 45–73 min). Assessment: SF-36	Significant improvement among 80% of women who completed the study on PCS (*p* = 0.03) and MCS (*p* = 0.0009).
Del Forno et al. 2020, Italy [30]	To assess the PFM physiotherapy, including Thiele massage and using 3-D/4-D transperineal ultrasound as biofeedback in women with deep infiltrating endometriosis and dyspareunia.	*n* = 10 women Mean age = 33.4 ± 9.2	PFM physiotherapy: information about PFM, Thiele massage, PFM exercises via 3-D/4-D ultrasound. 5 individual session of 30 min at 1,3,5,8,11 week Assessment: Gynecological examination, ultrasound examinations	PFM physiotherapy improves superficial (*p* = 0.0027) and deep (*p* = 0.0395) dyspareunia. Ultrasound was a valid visual feedback technique during PFM therapy.
Wurn et al. 2011, USA [31]	To assess the efficacy of manual therapy in dyspareunia and dysmenorrhea associated with endometriosis.	Retrospective analysis: *n* = 14 Mean age = 33.8 Prospective analysis: *n* = 18 mean age = 37.4	Site-specific manual therapy for 2 h/week for 5 months, then 4 h/day for 5 days. Assessment: FSFI, MPS	Improvement in each area of FSFI (*p* < 0.001) and dyspareunia (*p* < 0.001).
Muñoz-Gómez et al. 2023, Spain [37]	To analyze the effectiveness of a manual therapy protocol in relation to the pelvic pain, lumbar mobility, and clinical features related to quality of life and the emotional of women who suffer from pelvic pain due to endometriosis.	Manual therapy Group: *n* = 21 mean age = 34.85 ± 7.23 Placebo Group: *n* = 20 mean age = 37.4 ± 6.62	Manual therapy Group: 8 weeks, with one session for 30 min every 15 days, soft tissue and articulatory techniques included: (a) Occiput, atlas, and axis manipulation technique. (b) Thoraco-lumbar manipulation technique. (c) Global sacroiliac manipulation technique. (d) Abdominal mobilization technique. (e) Broad ligament mobilization technique. (f) Pelvic diaphragm release technique. (g) Sphenoid technique. (h) Fourth ventricle technique. Placebo Group: The participants received light contact on the same points and for the same amount of time as the experimental group with no intention to treat. Follow up: after intervention, one-month and six-month Assessment: EHP-30, SF-36, VAS, BDI-II, STAI, PGICS	There was a significant pain reduction in the manual therapy group at each point of follow-up (*p* < 0.001). There were no significant differences after the placebo group or at the follow-up (*p* > 0.05). Manual therapy Group significantly improved at one-month follow-up for the domains: pain (*p* < 0.001), control and powerlessness (*p* = 0.001), emotional wellbeing (*p* = 0.01), and EHP-30 total score (*p* < 0.001). Placebo group did not significantly improve any of the EHP-30 items after the intervention and at the follow-up.
Sillem et al. 2016, Germany [39]	To assess the efficacy of osteopathic diagnosis and treatment for women with chronic pelvic pain and painful pelvic floor muscle tightness not related to the menstrual cycle.	*n* = 28 women 14 women with endometriosis 14 women without endometriosis	1 to 24 (range 6) treatment sessions lasting 30 min: Sacroiliac joints, diaphragm, abdominal organ, temporomandibular joints and cervical spine mobilisation. PFM released by movement of the abdominal organ compartment in a cranial direction. Assessment: Gynecological examination, ultrasound examination, questions about satisfaction	10 of 14 women with endometriosis showed improvement in pain and pelvic floor muscle tightness after osteopathy sessions.
Del Forno et al. 2024, Italy [41]	To assess the effect of pelvic floor physiotherapy on urinary, bowel, and sexual functions in women with deep infiltrating endometriosis.	*n* = 31 Experimental Group: *n* = 17 Mean age = 32.5 ± 7.6 Control Group: *n* = 13 Mean age = 32.8 ± 6.7	Experimental Group: Information on pelvic floor anatomy and function, five individual 30 min PFM physiotherapy sessions at weeks 1, 3, 5, 8, and 11. Control Group: standard of care without receiving pelvic floor physiotherapy sessions. Assessment: ultrasound examinations, BFLUTS, KESS, FSFI	Improvement in superficial dyspareunia, chronic pelvic pain, and PFM relaxation were shown in Experimental Group. No statistically significance in urinary function, bowel and sexual function were found between groups (*p* > 0,05).

OMT- Osteopathic manipulative therapy; qol– quality of life; PFM– pelvic floor muscles; SF-36- Short Form Health Survey; PCS- Physical Component Summary; MCS- Mental Component Summary; FSFI- Female Sexual Function Index; MPS- Mankoski Pain Scale; EHP-30 - Endometriosis Health Profile Questionnaire; VAS– Visual Analogue Scale; BDI-II- Beck Depression Index; STAI - State Trait Anxiety Index; PGICS - Patient Global Perception of Change Scale; BFLUTS - Bristol Female Lower Urinary Tract Symptoms questionnaire; KESS - Knowles–Eccersley–Scott–Symptom questionnaire; FSFI - Female Sexual Function Index

### Electrophysical Agents in the Treatment of Endometrial Symptoms

Electrophysical agents (EPA) contain the areas of physiotherapy that uses physical factors like cold, heat, electrical stimulation in the treatment process. Electrotherapy has been employed for managing endometriosis pain, utilizing techniques such as electrical neuromuscular stimulation (NMES) [32], and transcutaneous electrical nerve stimulation (TENS) [34]. Women who received NMES for 30 min, 3 times a week for 10 weeks showed decreased of pain endometriosis symptom severity and better results in SF-36 (36-26-item short-form Health Survey) compared to those who did not receive therapy. It is worth emphasizing that NMES was the only form of therapy in this group and has independently demonstrated effectiveness [32]. EPA can also be used as a complement to other therapy. Mira et al. [34] investigated the effects of TENS as an adjunct to hormone therapy for controlling pelvic pain in deep endometriosis. Women who self-administered TENS at home twice daily for 20 min over 8 weeks in the parasacral region experienced significant reductions in chronic pelvic pain and deep dyspareunia, along with notable improvements in quality of life, compared to those solely receiving hormonal treatment [34]. Thabet et al. [35] evaluated the use of Pulsed High-Intensity Laser Therapy (HILT) in addition to hormonal treatment, confirming significant reductions in pain and enhanced quality of life compared to placebo [35].

Furthermore, better treatment outcomes were observed using virtual reality (VR) compared to a standard tablet. Merlot et al. [27] conducted a study where women managing endometriosis pain were treated with a specialized application incorporating auditory and visual sensations. Divided into two groups—one using regular tablets and the other utilizing a VR device—the women in the VR group reported significantly lower pain levels post-treatment compared to the control group [27]. Pain reduction was also noted following thermal biofeedback therapy incorporating relaxation techniques and breathing exercises. Additionally, women acquired pain management skills through this therapy, although caution is warranted in interpreting the results due to the small sample size of the study (*n* = 5) [33].

Electrophysical agents in studies presents effectiveness alone and combined with other treatment in improve quality of life and pain. The characteristics of studies describing the effectiveness of electrophysical agents in the treatment of endometriosis are presented in Table 5.

**Table 5:** Characteristics of studies describing the effectiveness of electrophysical agents in the treatment of endometriosis

Author, year, country	Purpose	Participants	Intervention	Results
Merlot et al., 2022, France, Canada [27]	To assess the effectiveness of digital therapeutics on pain in women with endometriosis.	*n* = 45 women Digital treatment Endocare Group: *n* = 23 Mean age = 32.2 ± 8.02 Control Group: *n* = 22 Mean age = 33.2 ± 8.12	Endocare Group: 20-minute treatment consisting of a combination of auditory and therapeutic procedures integrated in a 3D virtual reality environment. Control Group: 20-minute treatment with the same composition as the Endocare treatment but without any immersive effects of the virtual reality nor the auditory and visual stimuli. Follow up: at 15, 30, 45, 60, 240 min after treatment. Assessment: NRS	The mean reduction of pain was greater in the Endocare group (*p* < 0.001) than in the control group (*p* = 0.008). The mean maximum reduction in pain was 42% (95% CI 30.82–53.18) for Endocare and 22% (95% CI 15.38–28.53) for the control group.
Bi et al. 2018, China [32]	To assess the effect of NMES for the treatment of endometriosis-associated pain.	*n* = 154 women NMES Group: *n* = 83 Mean age = 31.6 ± 3.6, Control Group: *n* = 71 Mean age = 32.2 ± 4.1	NMES Group: Applied NMES on selected acupoints with 2–100 Hz for 30 min, 3x per week for 10 weeks. Control Group: No intervention. Assessment: NRS, ESSS, SF-36	Significant improvement on all scales NRS (*p* = 0.02), ESSS (*p* = 0.04), SF-26 (*p* < 0.01) in the NMES group. after 10 weeks.
Hawkins, Hart 2003, USA [33]	To assess the effectiveness of thermal biofeedback in the treatment of pain associated with endometriosis.	*n* = 10 women (5 completed)	Thermal biofeedback relaxation session for 15-min intervals with a 2-min break between, twice weekly for 2 months + daily home relaxation practice Assessment: WHYMPI	After the end of the therapy, the WHYMPI scores improved in 4/5 of the women and the quality of life improved significantly (*p* < 0.05).
Mira et al. 2020, Brazil [34]	To assess the effectiveness of complementary treatment using self-applied electrotherapy treatment for pain for deep infiltrative endometriosis.	*n* = 101 women Electrotherapy Group: *n* = 53 Mean age = 35.06 ± 6.17, Hormonal Group: *n* = 48 Mean age = 37.21 ± 6.51	Electrotherapy Group: hormonal treatment + TENS applied on S3-S4, frequency: 85 Hz; pulse duration: 75 ms; intensity options: 10, 20, or 30 mA, twice a day for 20 min for 8 weeks. Hormonal Group: Only hormonal treatment. Assessment: EHP-30, VAS, FSFI, DDS	Reduction of pain (36%), number of painful days (32.11%) and sexual function (9.16%) in the Electrotherapy Group, the level of dyspareunia and quality of life improved in both groups.
Thabet et al., 2018, Egypt, Saudi Arabia [35]	To assess the effectiveness of pulsed high-intensity laser therapy in women with endometriosis.	*n* = 40 women (24–32 years old) HILT Group: *n* = 20 Sham Group: *n* = 20	HILT Group: HILT, 120–150 ls pulse duration, duty cycle of 0.1%, frequency of 10–40 Hz for 20 min, 3 times per week for 8 weeks. Sham Group: sham laser treatment Assessment: PPi, PR, laparoscopy, EHP-5	Significant reduction in pain (+ 77.27%) and better quality of life (+ 73%), (*p* < 0.0001) in HILT Group.

NMES– neuromuscular electrical stimulation; NRS– Numerical Rating Scale; ESSS– Endometriosis Symptom Severity Score; SF-36–36-Item Short Form Health Survey; VAS– Visual Analogue Scale; TENS - transcutaneous electrical nerve stimulation; DDS– Deep Dyspareunia Scale; EHP-30– Endometriosis Health Profile; FSFI– Female Sexual Function Index; EHP-5– Endometriosis Health Profile, PPi– Present Pain Intensity; PR– Pain Relief scale; WHYMPI– West Haven-Yale Multidimensional Pain Inventory

### Acupuncture in the Treatment of Endometrial Symptoms

Acupuncture is a controversial therapy. Traditional Chinese therapy appeals to non-anatomical structures such as meridians. The analgesic effect of acupuncture may be due to the stimulation of the nerves in the epidermis which, by sending impulses through the spinal cord to the brain, stimulate opioid secretion and decreased pain levels [38].

Rubi-Klein et al. [36] conducted 10 therapeutic acupuncture sessions, with one group receiving authentic treatment and the other receiving a placebo. Following a crossover between the groups, better outcomes were noted in the authentic acupuncture group. Women reported significantly lower pain sensations post-therapy, accompanied by increased quality of life, compared to the placebo group [36]. De Sousa et al. [38] reached similar conclusions. They divided women with endometriosis into two groups, one group receiving acupuncture at appropriate sites and the other group receiving a placebo. After five sessions, marked improvements in pain and quality of life were observed in the authentic acupuncture group compared to the placebo group. These results persisted until the second month post-therapy [38]. Acupuncture also turned out to be more effective in treating menstruation pain in women with endometriosis, compared to women who used pain killers to reduce pain. Acupuncture was applied to specific points on each day of menstruation for 3 cycles. The effect lasted until the second cycle after the end of treatment [40].

Despite many controversies around acupuncture, studies have demonstrated its efficacy in pain relief. However, according to the European Society of Human Reproduction and Embryology, no definitive recommendation can be made regarding its use in women with endometriosis [50]. The characteristics of studies describing the effectiveness of acupuncture in the treatment of endometriosis are presented in Table 6.

**Table 6:** Characteristics of studies describing the effectiveness of acupuncture in the treatment of endometriosis

Author, year, country	Purpose	Participants	Intervention	Results
Rubi-Klein et al., 2010, Austria [36]	To assess the effectiveness of acupuncture as an additional pain treatment for endometriosis.	*n* = 101 women (83 completed) Exp.Gr: *n* = 47 (42 completed) Mean age = 34.8 Con.Gr: *n* = 54 (41 completed) Mean age = 32.5	Con. Gr: non-specific acupuncture Exp.Gr: verum-acupuncture Two units for 10 treatments sessions, twice a week, observation for at least two menstrual cycles, then cross-over. Assessment: SF-26, VAS, PDI	Verum acupuncture is effective in the treatment of pain (*p* < 0.0001) and increases the quality of life of patients.
de Sousa et al., 2016, Brazil [38]	To assess the effectiveness of an acupuncture protocol on chronic pelvic pain, dyspareunia, and quality of life in women with endometriosis.	*n* = 42 women Exp.Gr: *n* = 20 women Mean age = 30.45 ± 5.89 Con.Gr: *n* = 22 women Mean age = 31.14 ± 6.92	Con.Gr: Five session of acupuncture, needles inserted 3 cm apart from original points Exp.Gr: Five session of acupuncture, needles inserted in specific places Assessment: VAS, EHP-30	Acupuncture reduced pain in both groups (*p* = 0.004). However, 2 months after the therapy, the results were maintained only in the Exp.Gr.
Tian et al., 2021, China [40]	To assess therapeutic effect on dysmenorrhea in the patients with adenomyosis between acupuncture and ibuprofen sustained release capsules.	Acupuncture group: *n* = 20 Ibuprofen Group: *n* = 20	Acupuncture Group: Insertion of needles in specific acupoints during menstruation (every day of menstruation) and in non-menstrual period (twice a week) for 3 menstrual cycles. Ibuprofen Group: Oral Ibuprofen capsules, starting from 1st day of menstruation, 1 capsule twice a day for 5 days, for 3 menstrual cycles. Assessment: VAS, EHP-5, CMSS	Two menstrual cycles after treatment VAS score at the most painful time during menstruation was lower in Acupuncture Group (2.175 ± 1.507) than Ibuprofen Group (6.075 ± 0.748). CMSS and EHP-5 scores was lower in Acupuncture Group (*p* < 0.005).

SF-36–36-Item Short Form Health Survey; VAS– Visual Analogue Scale; PDI– Pain Disability Index; EHP-30– Endometriosis Health Profile; HRQOL - Health-Related Quality of Life; EHP-5– Endometriosis Health Profile-5; CMSS - COX menstrual symptom scale

### Diet and Cognitive Behavioral Therapy in the Treatment of Endometrial Symptoms

Endometriosis as a proinflammatory condition may be managed by diet. Some nutrients may decrease inflammatory factors, which can reduce pain [42]. Cirillo et al. [43] found a strong link between pain relief in endometriosis patients and Mediterranean dietary patterns. A individual Mediterranean diet shows promise for treating endometriosis-related symptoms and could be an effective long-term strategy for managing chronic pain alongside other nonmedical treatments [43]. Nodel et al. [42] confirmed that vitamin D supplementation in adolescents with surgically confirmed endometriosis significantly improved pelvic pain and catastrophic thinking, but these improvements were similar to those seen in the placebo group. Fish oil showed some improvement in VAS pain, but it was not statistically significant and was less effective than the other treatments. The study highlighted a strong placebo effect, indicating that participation in the study itself, rather than the supplements [42]. Van Haaps et al. [44] found that LOWFOOD diet or Endometriosis diet lead to reduced pain and improved quality of life for women with endometriosis after six months. Notably, those following the diet experienced less bloating and better quality of life in medical treatment and social support area [44].

The other treatment Cognitive Behavioral Therapy (CBT) may be beneficial for women with endometriosis due to the complex interplay between physical symptoms and mental health challenges associated with endometriosis. Donatti et al. [45] presented that CBT decreased depression from 64 to 12% in women, as well as stress prevalence decreased from 72 to 24%, and quality of live improved (*p* > 0.001) [45]. Wu et al. [46] assessed the impact of CBT and Tai Chi training on the quality of life of women who underwent surgery for endometriosis. Tai Chi training has shown effectiveness in reducing anxiety and stress, while the inclusion of CBT increased the positive effect on the quality of life and reduced depression [46]. In turn, Kold et al. [47] confirmed the effectiveness of mindfulness techniques, individual and group therapy. Women participating in the study significantly increased their quality of life and reduced pain associated with endometriosis.

Symptoms associated with endometriosis can also be effectively managed through psychological interventions and diet. A detailed description of the research can be found in Table 7.

**Table 7:** Characteristics of studies describing the effectiveness of diet and cognitive-behavioural therapy in the treatment of endometriosis

Author, year, country	Purpose	Participants	Intervention	Results
Nodler et al., 2020, USA [42]	To assess whether supplementation with vitamin D or ω-3 fatty acids remediates pain, changes frequency of pain medication usage, or affects quality of life in young women with endometriosis.	Vitamin D: *n* = 27 (23 completed) Mean age = 20.0 ± 2.7 Fish oil: *n* = 20 (17 completed) Mean age = 18.9 ± 3.1 Placebo: *n* = 22 (19 completed) Mean age = 20.1 ± 3.5	Vitamin D: 2000 IU vitamin D3 (cholecalciferol) daily Fish oil: 1000 mg fish oil [720 mg ω-3 fatty acids, including 488 mg EPA (20:5n–3) and 178 mg DHA (22:6n–3)] daily Placebo: taking white gelatin capsules with inert lactose powder. Assessment: baseline, 3 and 6 month after enrolment 128-item FFQ, SF-12, VAS, serum samples	VAS pain scores improved from baseline to 6 months in the placebo (5.5 to 4.6, *p* = 0.32), vitamin D (6.3 to 5.3, *p* = 0.15), and fish oil (5.6 to 5.1, *p* = 0.67). Participants in all 3 study arms demonstrated improvement in catastrophic thinking score, with a statistically significant mean score improvement from baseline to 6 months only in the vitamin D (25.3 to 20.8, *p* = 0.04).
Cirillo et al., 2023, Italy [43]	To assess the role of dietary changes according to the Mediterranean Diet pattern on pain perception in women with endometriosis and their relationship with oxidative stress.	*n* = 35 women with endometriosis (26 completed)	Each woman received an individually selected Mediterranean diet for 6 months. Assessment: blood sample, VAS, dyspareunia	Patients experienced reduced pain in dyspareunia (*p* = 0.04), non-menstrual pelvic pain (*p* = 0.06). Additionally, there was a significant positive correlation between lipid peroxidation and VAS non-menstrual pelvic pain.
Van Haaps et al., 2023, the Netherlands [44]	To assess the impact of the Low FODMAP diet and the endometriosis diet on endometriosis-related symptoms and quality of life.	Low FOODMAP diet: *n* = 22 Mean age = 36.9 ± 5.9 Endometriosis diet: *n* = 21 Mean age = 39.1 ± 15.8 Control: *n* = 19 Mean age = 37.6 ± 8.5	The Low FODMAP diet involves three phases: elimination of high-FODMAP foods for 6–10 weeks to reduce IBS symptoms, reintroduction of high-FODMAP foods one at a time to identify triggers, and personalization based on individual tolerance. In the endometriosis diet women avoid nutrients they noticed provoked or aggravated their endometriosis-related symptoms (e.g. red meat, gluten, cow milk, sugars). Control group did not received any diet. Assessment: VAS, EHP-30; GIQLI	All participants adhering to a diet reported significantly less deep dyspareunia and tiredness after adhering to the diet for 6 months compared to their baseline scores (*p* < 0.001). Participants adhering to the Low FODMAP diet reported significantly less dysuria (*p* = 0.015) and bloating (*p* < 0.001), whereas participants adhering to the endometriosis diet reported significant less bloating (*p* < 0.001) and tiredness (*p* = 0.002) after 6 months compared to their baseline scores. Participants in the control group reported no significantly different pain scores in endometriosis-related symptoms at 6 months follow-up.
Donatti, 2024, Brazil [45]	To assess the efficacy of CBT in enhancing coping strategies, alleviating depression, stress, reducing pain perception, and improving the quality of life for women suffering from endometriosis and chronic pelvic pain.	Experimental group: *n* = 25 Control group: *n* = 27	Experimental Group: 16 CBT session, 1session/week Control Group: no intervention Assessment: SF-36, Brief Cope, Beck Depression Scale, Lipp’s Adult Stress Symptoms Inventory, VAS	After 4 months, control group depression decreased to 55.56%, while the experimental group dropped to 12% post-CBT. For dysmenorrhea and chronic pelvic pain, post-intervention, likelihood of pain-free status was 14 times higher (*p* < 0.01). In quality of life, experimental group showed significant improvements in SF-36 scores, including physical functioning, role limitations, pain, general health, vitality, social functioning, emotional role limitations, and mental health.
Wu, 2022, China [46]	To assess whether usual care combined with CBT improves depression, anxiety, and stress in patients after surgery for endometriosis as compared to usual care alone.	Intervention group: *n* = 48 Control group: *n* = 48	Intervention group: 1 pre-surgery and 6 post-surgery CBT sessions in addition to their routine usual care. Control group: usual care - Tai Chi, 30 min/per day, 5 days a week Assessment: DASS-21	Depression, anxiety, and stress of the case group and the control group were decreased as compared to baseline (*p* < 0.001). Usual care plus CBT significantly increased the number of females with no symptoms of depression (*p* = 0.0356). Usual care plus CBT significantly decreased the number of females with symptoms of extremely severe anxiety (*p* = 0.035).
Kold, 2012, Denmark [47]	To assess the feasibility of mindfulness approach in patients with chronic pain secondary to endometriosis.	*n* = 10 Median age = 23	5 individual and 5 group session of mindfulness, visualization, psycho-education and group support methods. Assessment: SF-36, EHP-30	Bodily pain significantly and consistently improved from pre- to post-intervention and follow-up measures (*p* < 0.05). The work life scale showed significant improvement on all measurement points. Pain decreased from 52.53 to 28.18 (*p* < 0.001).

SH-12– Short form 12; FFQ - Food Frequency Questionnaire; VAS– visual analogue scale; EHP-30 - Endometriosis Health Profile; GIQLI– Gastro-intestinal health; CBT– Cognitive behavioural therapy; SF-36 - The Short Form Health Survey; DASS-21 - Depression anxiety and stress scale

## Discussion

The aim of this review was to outline the most prevalent physiotherapeutic and non-medical approaches utilized in addressing symptoms linked with endometriosis and to assess their efficacy.

Endometriosis is often associated with chronic pelvic pain [51], frequently intensifies during menstruation [12]. Pain prompts individuals to adopt antalgic postures, and poor body posture, in turn, fosters myofascial disorders, such as muscle shortening, heightened tension, and consequently, weakness [12, 52]. Women may present Myofascial Trigger Points in the pelvic floor muscles as well as devious locations, complicating their identification. Trigger Points are a hypersensitive spot in the taut band and stimulation of this point cause referred pain [53]. These Points can disrupt both motor and autonomic function, disrupting the function of visceral organs [54]. Prolonged muscle tension causes muscle ischemia, worse trophic, stimulating pain receptors [55], which in turn leads to pelvic floor dysfunction [15]. Nevertheless, theories regarding trigger points are controversial [56]. Studies showed, that many women resign from physical activity due to pain [57]. Pain induces reduced activity, which precipitates trophic alterations in soft tissues, compromising their function, thereby weakening motor control in the lumbopelvic region, amplifying pain, and curtailing activity and social engagement [51]. The phenomenon of central sensitization is also often observed in women with endometriosis, which may be related to a lower response to treatment [58]. Nociceptive neurons in the dorsal horn of the spinal cord increase their excitability by repeated exposure to noxious stimuli, such as damage. Long-term irritation of nociceptive neurons causes a reduced pain threshold and an increased response to pain. Long-term pain also causes changes in the activity and structure of the brain, leading to changes in the processing of pain and sensory impulses. In addition, changes are also observed in the hypothalamic-pituitary-adrenal axis, which is also responsible for pain modulation [59].

Endometriosis exerts a profound impact on women’s lives, manifesting in reduced quality of life. Endometriosis-associated conditions, including sleep disturbances, fatigue, depression, anxiety, infertility, diminished productivity, and sexual dysfunction, impinge upon various aspects of life. Literature review and multivariate analysis of the impact of endometriosis on life performed by Missmer et al. [60] showed that endometriosis affects educational achievements, social, family and emotional life, and mental health [60]. To reduce the negative impact of the disease on the quality of life, it’s crucial to detect endometriosis early and initiate treatment promptly. Pharmacological therapies are commonly used for endometriosis symptoms, however may be associated with sleep disturbances, hot flashes, vaginitis, headaches, nausea and decreased bone density [61]. Pharmacotherapy typically results in a reduction of pelvic pain by approximately 2 points on a 10-cm visual analogue scale after 3 months [62]. However, despite the many side effects associated with pharmacological treatment, physiotherapy appears to offer an equally effective alternative for alleviating symptoms linked with endometriosis. Physiotherapeutic interventions employed in managing endometriosis symptoms encompass physical therapy, comprising exercises [25], aerobic training [26], yoga [22] and relaxation techniques, such as stretching, breathing [24] and progressive muscle relaxation [28]. Physical activity seems to be an effective, non-invasive method of alleviating the side effects of medications, delaying the decline in bone density, increasing the quality of life, and reducing pain. Physical therapy proves efficacious in reducing stress, anxiety, and normalizing cortisol levels [24]. Pain, dysmenorrhea and dyspareunia may be also treated by manual therapy [29–31, 37, 39]. Through myofascial connections, tensions can be transferred to other areas of the body, while inflammation and an increased number of inflammatory mediators in the pelvic organ area can contribute to myofascial disorders, intra-organ movement and vascular drainage [63]. Visceral therapy improved physical and mental function among 80% of women with endometriosis [29]. Transvaginal manual therapy relaxes muscles and restores normal pelvic tone, consequently reducing dyspareunia [30]. Adhesions commonly occurre with endometriosis and can be identified by physiotherapists; specialized techniques enable the detachment of adhesive crosslinks and alleviate pain during menstruation and intercourse [31]. Specialists may also use transperineal ultrasound to evaluate pelvic floor muscle functioning and localize muscles dysfunction [30]. Other complementary treatment for symptoms associated with endometriosis may be electrotherapy, exactly transcutaneous electrical nerve stimulation (TENS), which reduce pain. Studies suggest that TENS reduced chronic pelvic pain in VAS scale for approximately 2.55 points, whereas hormonal treatment alone reduced pain for approximately 0.27 points in VAS scale [34]. Positive outcomes have also been observed in studies on electrical neuromuscular stimulation (NMES); after 5 weeks of NMES treatment, pain decreased by approximately 1.4 points on a scale ranging from 0 to 10 [32]. Besides electrotherapy, epth are important [35]. Virtual reality may also prove to be a helpful technique in modern physiotherapy treatment aimed at better pain modulation [30]. Acupuncture is more and more often used as a therapy for gynecological disorders, despite the controversies. It demonstrates positive effects in women with endometriosis, reducing chronic pelvic pain by 66% and dyspareunia by 65%, with the effects persisting for at least 2 months post-acupuncture therapy [38]. Acupuncture exhibited a superior analgesic effect compared to Ibuprofen during menstruation in women with endometriosis [40]. Endometriosis, a proinflammatory condition, may be managed through dietary interventions, such as the Mediterranean diet, which has been linked to pain relief in patients [42]. Vitamin D supplementation and fish oil showed some benefits, though a strong placebo effect was noted [42]. The LOWFOOD diet also reduced pain and improved quality of life, particularly in reducing bloating and enhancing social support [44]. Cognitive Behavioral Therapy (CBT) has proven effective in reducing depression, stress, and improving the quality of life for women with endometriosis [45]. Additionally, Tai Chi and mindfulness techniques, both individual and group therapy, have shown significant benefits in managing anxiety, stress, and pain associated with endometriosis [46, 47].

Our review has its limitations. Firstly, many of the studies included had small sample sizes, and participant selection was not always heterogeneous, thus caution should be exercised in interpreting the results. Often, the research included women with severe endometriosis, which may not necessarily reflect outcomes in women with milder symptoms. Another constraint is the lack of validation of questionnaires for specific populations. Additionally, a considerable number of participants were lost during the study and follow-up. Not all studies were randomized, and some lacked proper controls. Short follow-up periods hindered the determination of long-term therapy effects. Furthermore, publications were restricted to those available in Polish and English. It’s important to note that specific criteria regarding the duration and type of research were not uniformly applied, which could influence the findings. Nonetheless, this allowed us to identify common non-medical methods for treating endometriosis and pinpoint areas requiring further investigation.

In conclusions, it is worth add physiotherapy methods in the reduce of symptoms of endometriosis. Physical activity, manual therapy, electrophysical agents, acupuncture, diet and cognitive behavioral therapy showed no negative side effects and reduced pain, what improved the quality of life and reduced the perceived stress.

## Electronic Supplementary Material

Below is the link to the electronic supplementary material.

Supplementary Material 1

Supplementary Material 2

## Data Availability

not applicable.
