# Supplementary material for: Holistic Approaches in Endometriosis - as an Effective Method of Supporting Traditional Treatment: A Systematic Search and Narrative Review
Source: Reprod Sci. 2024 Jul 23;31(11):3257–74. doi: 10.1007/s43032-024-01660-2 (PMC11527925; doi:10.1007/s43032-024-01660-2)
Supplement: Supplementary file 2 — Supplementary Material 2 [file 43032_2024_1660_MOESM2_ESM.pdf]

Physiotherapy in endometriosis - as an effective method of supporting traditional treatment: a systematic review.

To enable PROSPERO to focus on COVID-19 submissions, this registration record has undergone basic automated checks for eligibility and is published exactly as submitted. PROSPERO has never provided peer review, and usual checking by the PROSPERO team does not endorse content. Therefore, automatically published records should be treated as any other PROSPERO registration. Further detail is provided here.

Citation

Agnieszka Mazur-Bialy, Sabina Tim. Physiotherapy in endometriosis - as an effective method of supporting traditional treatment: a systematic review.. PROSPERO 2023 CRD42023389400 Available from:
https://www.crd.york.ac.uk/prospero/display_record.php?ID=CRD42023389400

Review question

1. What are the most common physiotherapeutic methods used in the treatment of symptoms associated with endometriosis?

2. Which symptoms associated with endometriosis are most often treated by physiotherapists?

3. Which physiotherapeutic methods are the most effective in the relief of symptoms associated with endometriosis?

Searches

Sources: MEDLINE-Pub Med, Web of Science, EMBASE

language: english and polish

publication date: no time limit

Types of study to be included

no restrictions

Condition or domain being studied

Endometriosis is one of the gynecological diseases where it is observed the presence of estrogen-sensitive, living tissue of the endometrium outside the uterus.Women may not feel any discomfort or report painful periods, bleeding between periods, urinary problems, painful intercourse, painful bowel movements, diarrhoea or abdominal pain unrelated to menstrual pain. Endometriosis is often associated with chronic pelvic pain, which change posture and lead to weakening the trunk muscles. Incorrect body posture will also have an effect on the pelvic floor. Among women with endometriosis, hyperactivity of the pelvic floor and the presence of trigger points are observed. Endometriosis significantly affects physical and mental health and quality of life.

Participants/population

Inclusion criteria: women, pain related to endometriosis, endometriosis, aged above 16 years old

Exclusion criteria: pain related with cancer, gynecological cancer, aged below 16 years old

Intervention(s), exposure(s)

any physiotherapeutic intervention (exercise, manual therapy, physical therapy)

Comparator(s)/control

no intervention, placebo

Context

Studies on women with endometriosis and its symptoms and containing physiotherapeutic methods treating these symptoms.

Main outcome(s)

Find the physiotherapuetic methods treating symptoms of endometriosis.

Assess which methods are the most effective in treating symptoms of endometriosis measured.

Measures of effect

Reduces of symptoms through questionnaires or subjectively.

Additional outcome(s)

none

Data extraction (selection and coding)

Researchers will individually search the databases according to search strategies. The found records will be saved in Excel. After completing their search, the authors will compare the results obtained and the Risk of Bias will bed done. The study protocol will be shown on the PRISMA diagram.

Risk of bias (quality) assessment

The Cochrane Risk of Bias 2 tool assesses the quality of randomized studies, while the ROBINS-I analysis allows the quality of non-randomized studies or interventions.

Strategy for data synthesis

A critical and detailed analysis of the included publications will be performed to determine the effectiveness of physioterapeutic methods used in the treatment of symptoms associated with endometriosis. Studies with high risk will be ecxluded from analysis.

Analysis of subgroups or subsets

No subgroups are planned.

Contact details for further information

Sabina Tim

sabina.tim@doctoral.uj.edu.pl

Organisational affiliation of the review

Department of Biomechanics and Kinesiology, Faculty of Health Science, Jagiellonian University Medical College, Krakow, Poland

Review team members and their organisational affiliations

Professor Agnieszka Mazur-Bialy. Department of Biomechanics and Kinesiology, Faculty of Health Science, Jagiellonian University Medical College, Skawińska 8, 31-066 Krakow, Poland

Ms Sabina Tim. Department of Biomechanics and Kinesiology, Faculty of Health Science, Jagiellonian University Medical College, Skawińska 8, 31-066 Krakow, Poland

Type and method of review

Systematic review

Anticipated or actual start date

08 January 2023

Anticipated completion date

30 June 2023

Funding sources/sponsors

none

Conflicts of interest

Language

English

Country

Poland

Stage of review

Review Ongoing

Subject index terms status

Subject indexing assigned by CRD

Subject index terms

Endometriosis; Female; Humans; Medicine; Pelvic Pain; Physical Therapists; Physical Therapy Modalities

Date of registration in PROSPERO

15 January 2023

Date of first submission

04 January 2023

Stage of review at time of this submission

The review has not started

Stage	Started	Completed
Preliminary searches	No	No
Piloting of the study selection process	No	No
Formal screening of search results against eligibility criteria	No	No
Data extraction	No	No
Risk of bias (quality) assessment	No	No
Data analysis	No	No

The record owner confirms that the information they have supplied for this submission is accurate and complete and they understand that deliberate provision of inaccurate information or omission of data may be construed as scientific misconduct.

The record owner confirms that they will update the status of the review when it is completed and will add publication details in due course.

Versions

15 January 2023

15 January 2023
